# The Competitive Influences of Perceptual Load and Working Memory Guidance on Selective Attention

**DOI:** 10.1371/journal.pone.0129533

**Published:** 2015-06-22

**Authors:** Jinfeng Tan, Yuanfang Zhao, Lijun Wang, Xia Tian, Yan Cui, Qian Yang, Weigang Pan, Xiaoyue Zhao, Antao Chen

**Affiliations:** 1 Key Laboratory of Cognition and Personality of Ministry of Education, Faculty of Psychology, Southwest University, Chong Qing, China; 2 Key Laboratory for NeuroInformation of Ministry of Education, School of Life Science and Technology, University of Electronic Science and Technology of China, Chengdu, China; University of Electronic Science and Technology of China, CHINA

## Abstract

The perceptual load theory in selective attention literature proposes that the interference from task-irrelevant distractor is eliminated when perceptual capacity is fully consumed by task-relevant information. However, the biased competition model suggests that the contents of working memory (WM) can guide attentional selection automatically, even when this guidance is detrimental to visual search. An intriguing but unsolved question is what will happen when selective attention is influenced by both perceptual load and WM guidance. To study this issue, behavioral performances and event-related potentials (ERPs) were recorded when participants were presented with a cue to either identify or hold in memory and had to perform a visual search task subsequently, under conditions of low or high perceptual load. Behavioural data showed that high perceptual load eliminated the attentional capture by WM. The ERP results revealed an obvious WM guidance effect in P1 component with invalid trials eliciting larger P1 than neutral trials, regardless of the level of perceptual load. The interaction between perceptual load and WM guidance was significant for the posterior N1 component. The memory guidance effect on N1 was eliminated by high perceptual load. Standardized Low Resolution Electrical Tomography Analysis (sLORETA) showed that the WM guidance effect and the perceptual load effect on attention can be localized into the occipital area and parietal lobe, respectively. Merely identifying the cue produced no effect on the P1 or N1 component. These results suggest that in selective attention, the information held in WM could capture attention at the early stage of visual processing in the occipital cortex. Interestingly, this initial capture of attention by WM could be modulated by the level of perceptual load and the parietal lobe mediates target selection at the discrimination stage.

## Introduction

Moment to moment, our sensory system is bombed by a tremendous amount of information. Because of the limited processing ability, the brain can only process small part of them at one time. Accordingly, we had developed the selective attention mechanisms to focus on the information relevant to our current goals. Some studies suggested that a major determinant of selective attention is the level of perceptual load in a relevant task [1,2]. Specifically, the high perceptual load in a relevant task facilitates the filtering to irrelevant information. On the other hand, WM was also found to influence the selective attention. When a template being retained in WM matched the target of visual search task, the attention can be automatically guided to the location of the target, and the search efficiency was accordingly improved [3,4,5].

Research on the role of perceptual load in selective attention was promoted by the hypothesis that perception has limited capacity but processes all stimuli in an automatic mandatory fashion until it runs out of capacity [6]. Tasks with high perceptual load engaging full capacity will simply leave no capacity for perception of irrelevant distractor. In contrast, in situations of low perceptual load, spare capacity remaining beyond the task-relevant processing spills over involuntarily to irrelevant distractor processing [7]. Evidences in support of the perceptual load theory have been found in many studies with response competition paradigm [1,2,6,8], attentional capture paradigm [9,10] and inattention blindness paradigm [11]. These behavioral studies suggested that interference from task-irrelevant distractors is reduced when the target is under high perceptual load. At the electrophysiological level, perceptual load has been shown to affect task-irrelevant stimulus processing at the sensory-perceptual level, as indexed by the early visual P1 [12–14] or N1 component [13,15]. These ERP results thus provide direct electrophysiological support for proposals that linking perceptual load to early attentional selection in visual processing. The timing of this effect may vary from P1 to N1, which depends on whether the absolute perceptual load level exceeds the capacity limits of the P1 component or not.

WM representation is also believed to influence selective attention. The relationship between visual selection and WM has been highlighted by several lines of research. At the behavioural level, the deployment of attention in visual space can be automatically biased to stimuli matching the content of WM [16–20], which is consistent with the biased competition model. Subsequent works by Soto and colleagues [18,21,22] also demonstrated that the WM item reappearing in the search display did affect the direction of the first saccade. By signal-detection analyses, Soto et al. (2010) [23] and Pan et al. (2012) [24] proposed that the reappearance of WM item in the visual array can increase perceptual sensitivity to the memory-matching item. These findings might suggest that reentrant feedback from WM can affect early stages of perceptual processing. Intriguingly, this speculation was demonstrated by a recent ERP study [25], in which increased P1 amplitude was observed when WM content reappeared in search task. More indirect evidences were from brain imaging studies. Single-cell recording studies demonstrated that the matching between an external stimulus and a WM representation was associated with increased responses in the inferior and medial temporal cortex [26,27]. Other neuroimaging studies using similar paradigms with human participants suggested that the reappearance of a stimulus held in WM enhanced activity in the superior frontal gyrus, midtemporal, and occipital areas that are known to encode the prior occurrence of visual stimuli [28–30].

Hence, both perceptual load and WM representation are able to influence the visual attention selection. High perceptual load reduces perceptual sensitivity to task irrelevant stimulus, rather than affecting response criterion or bias [7,31]. Nevertheless, matching to the contents of WM increased perceptual sensitivity even under conditions that minimized competition for selecting the target [23]. An intriguing and unsolved issue is how the brain drives ongoing behavior when the content held in WM happens to match the distractor of visual search task at high perceptual load. According to the perceptual load theory, when the perceptual load in processing task-relevant stimuli is sufficiently high to exhaust all the perceptual capacity, the interference from distractor will be eliminated. Whereas, the biased competition model will lead to an opposite prediction: because of the attention guidance from WM, the interference from the distractor matching the WM item will resist even at high perceptual load. To investigate the distinct and competitive influences of perceptual load and WM representation on selective attention, and the corresponding neural dynamics, we integrated the experimental paradigms from the two lines of perceptual load and WM guidance studies with the ERP recording of high temporal resolution. The present design requires participants to keep an object in WM and perform a search task concurrently [25]. Meanwhile, to enable perceptual load to come into effect, we manipulated the perceptual level of visual search task. Previous work has shown that the earliest WM guidance effect was observed within the P1 time range [25] and this effect has been localized to occipital area by using sLORETA. The present study sought to extend our previous work to investigate whether and when the earliest WM guidance could occur in the high perceptual load level of search task.

## Materials and Methods

### Ethics Statement

Approval of the study was made by the Human Research Ethics Committee of the Southwest University of China. Informed consent was obtained from all the participants. All data underlying the findings are fully available without restriction. Data are available from DRYAD using the DOI.

### Participants

Two groups of healthy participants, with normal or corrected-to-normal vision, were recruited from the Southwest University and paid for participation. The WM group consisted of 22 participants and data from two participants were excluded because of technical problems and excessive eye blinks, and data from other two participants were also excluded from further analyses because of overmuch bad electrodes. The remaining 18 participants (10 females) were between 18 and 23 years of age (M = 21 years). The priming group included 22 participants and data from two participants were excluded from further analyses because of excessive eye blinks. The remaining 20 participants (10 females) were between 19 and 26 years of age (M = 21 years). All were right-handed healthy undergraduates.

### Stimuli and procedure

**Fig 1:**
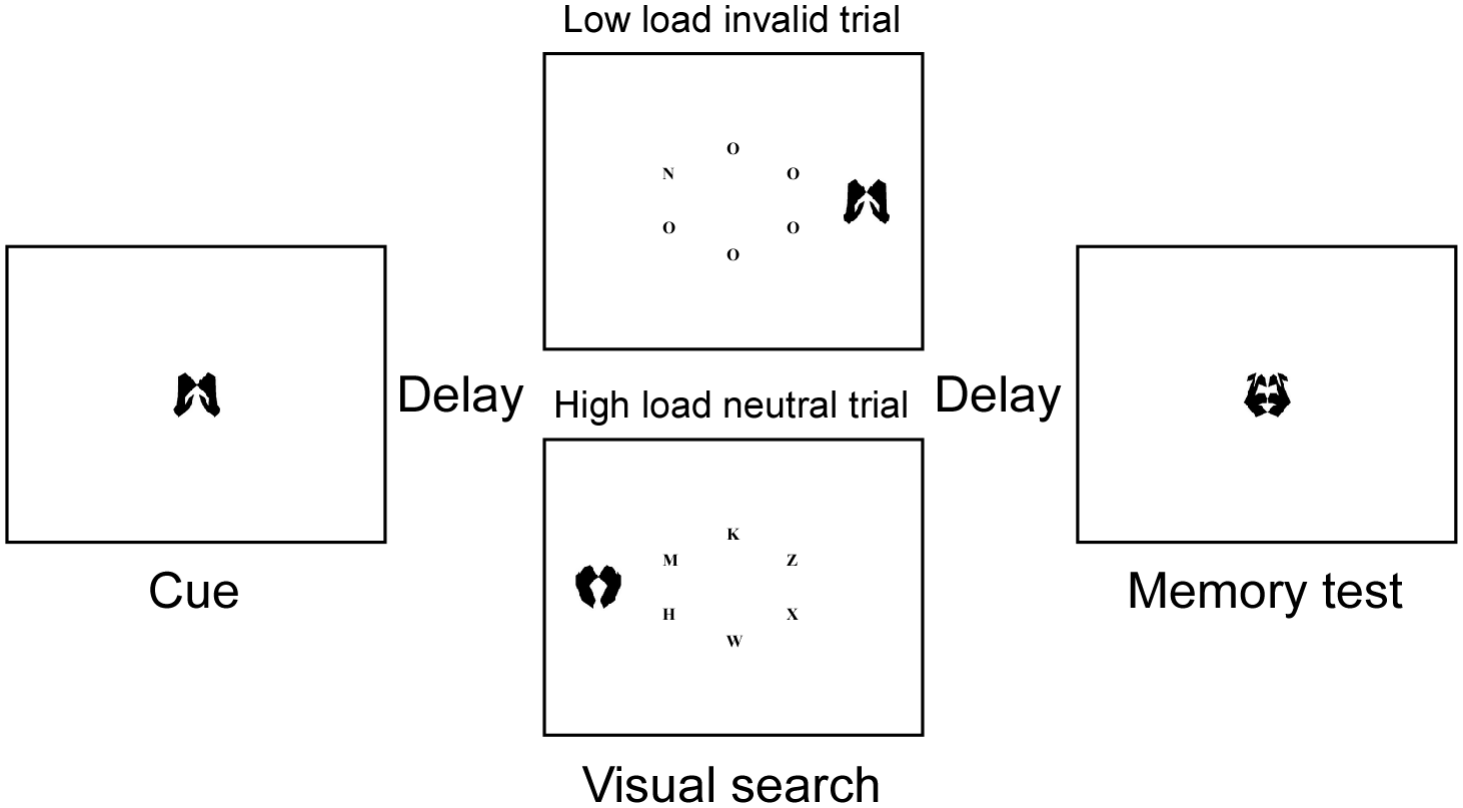
Examples of the different types of trial.

E-prime Software (Psychology Software Tools, Inc. Pittsburgh, PA) was used to present the stimuli and record the behavioral responses of the participants. The stimuli (letters and images) were displayed on a 17-in computer screen placed about 60 cm away from the participants. As depicted in Fig 1, the stimuli were black and presented on white background. Trials began with a fixation (a red asterisk) in the center of screen for 600 ms. Then, the cue was displayed at the fixation location. In the WM group, the cue appeared only once and presented for 1000 ms, during which participants had to memorize the image cue for the subsequent memory test. In the priming group, the cue appeared twice, the first time for 150 ms and the second time for 700 ms, with a blank interval of 150 ms between them [28,32]. Participants were instructed to perceptually compare the two instances of the cue and to withhold their response to the subsequent search display whenever the second presentation of the cue differed from the first presentation (20% likelihood throughout the experiment). After a 600–800 ms blank screen, a visual search task began and at longest lasted for 2000 ms, during which the participants needed to conduct the visual search and respond accordingly. Finally, a second fixation (across) jittered for 300–500 ms, followed by a memory probe image for 2000 ms, during which participants completed a memory test.

In the visual search task, participants were asked to search letter circle for a target letter (either X or N) as quickly as possible. The stimulus display consisted of a centrally presented 1.41° radius imaginary circle of six letters (each subtending 0.15° by 0.15°), plus a peripheral meaningless picture (each subtending 3.1° by 3.1°) as the distractor, which was presented 1.75° to the left or right of the center of the circle. In the high perceptual load condition, the nontarget letters in the circle were five angular letters (K, Z, W, M, and H) placed randomly around the circle, and in the low perceptual load the nontarget letters were five Os. Participants would press the “1” key of the keyboard if the target was N using their left middle finger and pressing the “0” key if the target was X using their right middle finger. The visual search trial terminated once a response was made. There were two conditions in which the relation between the cue and the search display was varied. On invalid cueing trials (50% likelihood throughout the experiment), the cue reappeared in the search display as a distractor. On neutral trials (50% likelihood throughout the experiment), the cue did not reappear in the search display. In the WM group, participants were instructed to hold the cue image in memory while performing the visual search task. In the priming group, participants should not make a response to the search display when the two cues were different. They were explicitly instructed to ignore the distractor and respond as quickly as they could while not sacrificing accuracy.

Upon the appearance of the memory probe, in the WM group, participants were instructed to indicate whether or not the probe image was the to-be-remembered image presented at the start of the trial. Subjects indicated either a same or different response by pressing the “same” (“Q”) or “different” (“O”) keys on the computer keyboard with the index fingers of their left hand and right hand, respectively. On half trials, the memory test was identical to the memory set, on the other half, it was not. The memory test terminated once a response was made. In the priming group, participants should not make any response.

In the WM group, each participant completed 384 trials in 8 blocks. After each block of 48 trials, the participants had a short break (1 min), during which they were told to relax. In the mere repetition group each participant completed 480 trials in 8 blocks. After each block of 60 trials, the participants had a short break (1 min). All task parameters including load, target position, target identity, distractor condition, and response-sides were pseudo-randomized and counterbalanced across subjects. One training block of 24 trials was run prior to the start of the main experiment.

### Electroencephalogram data processing

Brain electrical activity was recorded from 64 scalp sites using tin electrodes mounted on an elastic cap (Brain Products GmbH, Germany). All channels were referenced online to a channel located on FCz. The vertical electrooculogram (EOG) was recorded with electrodes placed below the right eye, while the horizontal EOG was recorded with electrodes placed on the right side of the right eye. Inter-electrode impedance was maintained below 5 kΩ. The data were rereferenced to the infinity zero reference (IR) using the software REST (Reference Electrode Standardization Technique; free software REST can be found at www.neuro.uestc.edu.cn/rest). REST is a novel method that builds a bridge between a physical reference and the theoretical neutral reference at an infinity point and the newly developed IR could provide increased accuracy [32–35]. The electroencephalogram (EEG) and EOG were amplified using a 0.01–30 Hz band pass, and continuously sampled at 500 Hz/channel for offline analysis. Eye movement artifacts (blinks and eye movements) were rejected offline. An artifact criterion of ± 80 μVwas used at all of the other scalp sites to reject trials with excessive electromyographs (EMGs) or other noise transients.

Further EEG analyses examined the P1 and N1 components in the stimulus-locked waveform. In the WM group, only trials with correct responses for both tasks were used in the analyses. The averaged epoch for the ERP elicited by the visual search trials was 800 ms, including 200 ms before trial onset and 600 ms after trial onset. Based on previous researches [36–38] and the scalp topography distributions of the difference waves in the present study, the following scalp region-of-interests (ROIs) and time windows were defined. We chose the left occipito-parietal (PO3, PO7, and O1) and right occipito-parietal (PO4, PO8, and O2) scalp regions. We defined the time windows of P1 (80–120 ms) and N1 (150–190 ms) both in the WM group and the mere repetition group. The ERP mean amplitude measures for P1 and N1 were then submitted separately to four-way ANOVAs that examined group (WM, priming) × perceptual load (low, high) × validity (invalid, neutral) × hemisphere (ipsilateral, contralateral). Contralateral waveforms were constructed by averaging the left hemisphere electrodes for right hemifield distractors and right hemisphere electrodes for left hemifield distractors. Ipsilateral waveforms were constructed by averaging the right hemisphere electrodes for right hemifield distractors and left hemisphere electrodes for left hemifield distractors [39,40].

On the basis of the ERP components’ scalp topographies, sLORETA was used to localize the cortical generators of P1 and N1 for WM group. sLORETA provides a unique standardized distributed linear solution to the inverse problem based on the neurophysiological assumption that the activities of neighboring cortical areas are coherent. Accordingly, it estimates multiple simultaneously active sources, thus avoiding the difficulties on estimating the number and position of the underlying dipoles. sLORETA uses a three-shell spherical head model co-registered to the MNI152 template, restricting solution space to the gray matter and hippocampus. The solution space is further partitioned in 6239 voxels at 5-mm spatial resolution. With a transformation matrix, the standardized current density at each voxel is calculated, forming a voxel-based whole-brain (grey matter and hippocampus) sLORETA image. LORETA methods (including LORETA, sLORETA, eLORETA) have received considerable validation from studies combining them with other more precise localization methods such as functional Magnetic Resonance Imaging.

For the present study, the transformation matrix was calculated with a regularization parameter (smoothness) corresponding to a signal-to-noise ratio (SNR) of 10. Then the distributed neural activities for P1 (from 80 to 120 ms) and N1 (from 150 to 190 ms) were estimated individually for each condition. The resulted individual current density images for each condition were averaged across participants to obtain the final grand mean P1 and N1 sLORETA images. Voxels of the grand mean sLORETA images that showed maximal activities in each condition for P1 and N1 were located in anatomical regions and Brodmann areas (BAs).

## Results

### RT data

**Fig 2:**
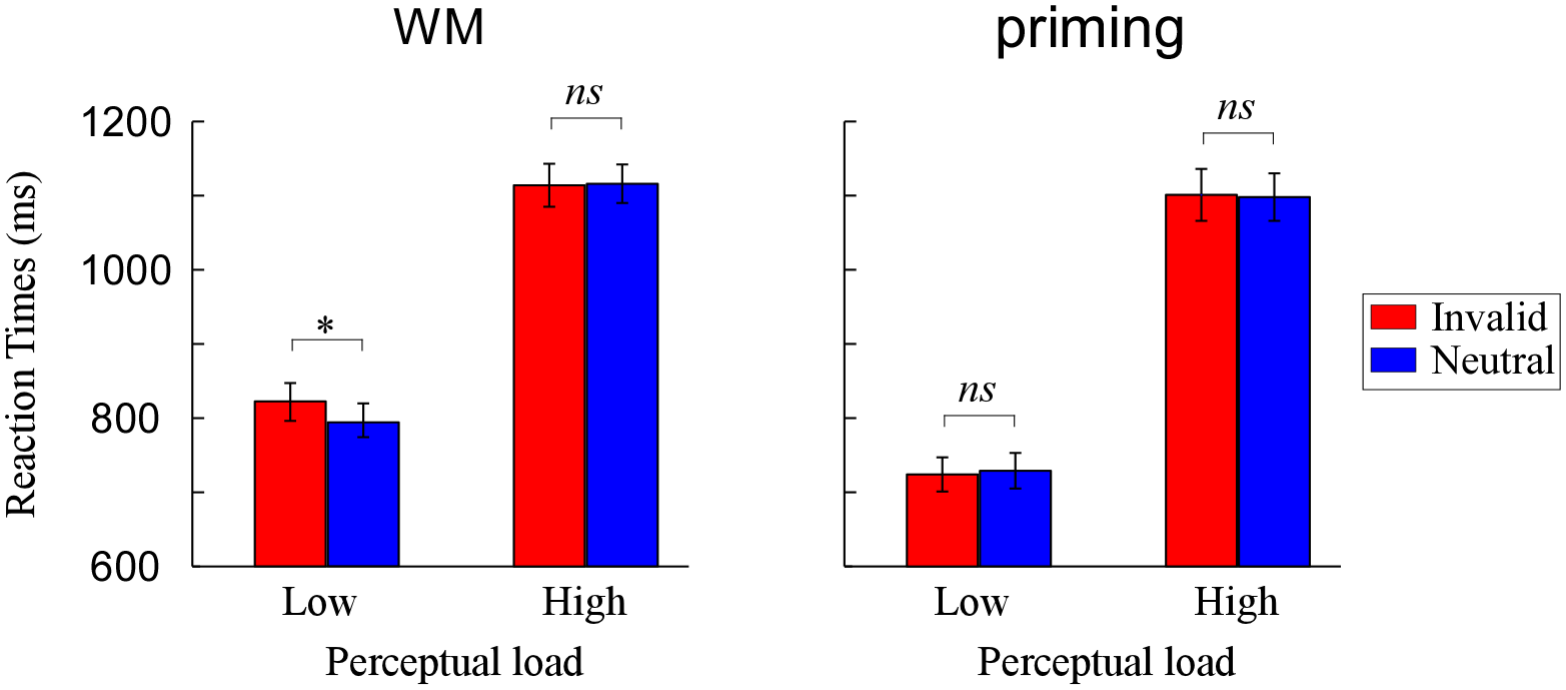
Mean reaction times (RTs) as a function of perceptual load and validity when the cue was held in WM and when it was merely identified. *p < .05, **p < .01, ***p < .001.

Performance was accurate in both the search task (mean 97% correct across WM group and mean 96% across priming group) and memory task (93% correct, WM group only). In the priming group, response on search trials were withheld as instructed (mean 97% correct). We analyzed mean RTs of the correct responses in all groups, using a 2 (group: WM, priming) × 2 (load: low, high) × 2 (validity: invalid, neutral) repeated-measures ANOVA. The results indicated a significant main effect of perceptual load [*F* (1, 36) = 635.19, *p* < 0.001], with slower RTs on high perceptual load than on low perceptual load trials. There was also an interaction effect between group and load [*F* (1, 36) = 5.98, *p* < 0.05]. The load effect was significant both in the WM group [*F* (1, 36) = 246.01, *p* < 0.001] and the priming group [*F* (1, 36) = 403.45, *p* < 0.001]. Crucially, the interaction between group, perceptual load and validity was significant [*F* (1, 36) = 3.38, *p* < 0.05]. A further breakdown of the interaction showed a reliable interaction effect between perceptual load and validity in the WM group [*F* (1, 17) = 7.18, *p* < 0.05] and not in the priming group [*F* < 1]. Specifically, in WM group, the validity effect on RTs (invalid minus neutral) was significantly present at low perceptual load [*F* (1, 17) = 13.87, *p* < 0.01], not at high perceptual load [*F* < 1]. Fig 2 depicts this pattern of performance.

### ERP measures

#### P1 component

**Fig 3:**
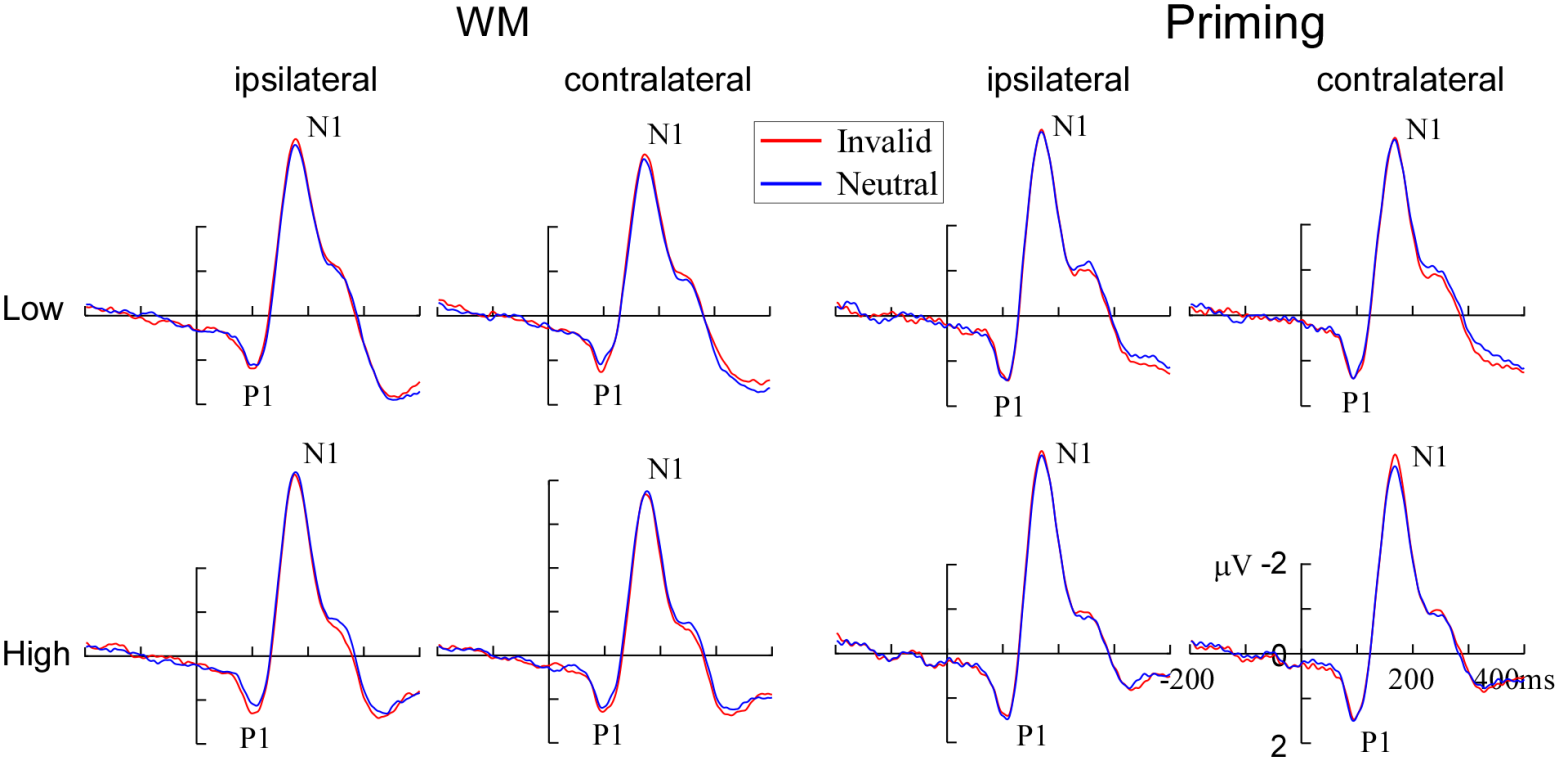
The validity effects on the P1 and N1 component under the low perceptual load (upper panels) and the high perceptual load (lower panels) conditions when the cue was held in WM (left panels) and when it was merely identified (right panels). Data were also averaged across memory-matching stimuli at ipsilateral vs. contralateral electrode sites. The left occipito-parietal electrodes include O1, PO3 and PO7 and the right occipito-parietal electrodes include O2, PO4 and PO8.

**Fig 4:**
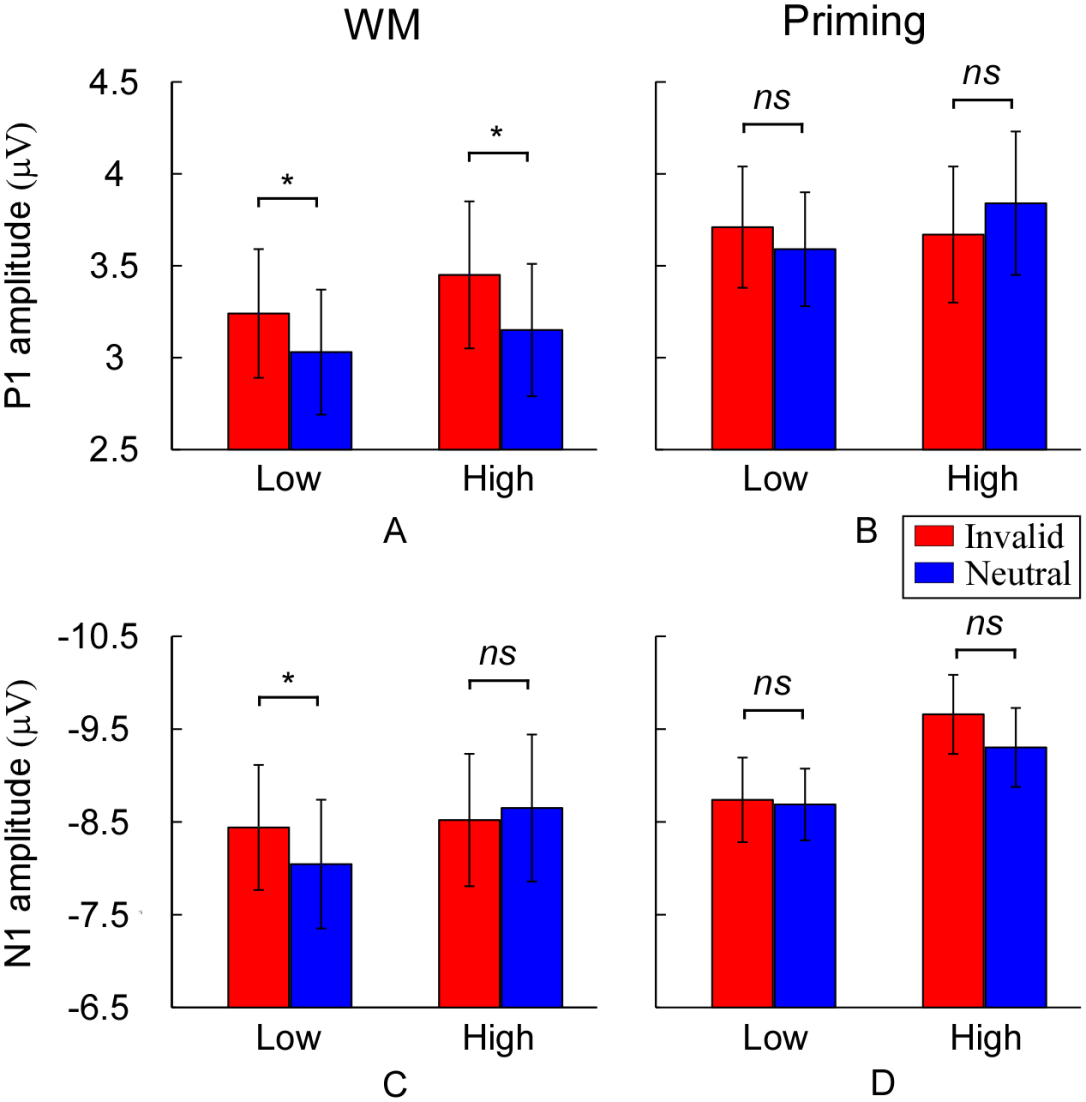
The mean amplitude for the P1 (A and B) and N1 component (C and D) as a function of perceptual load and validity of the stimuli across WM group and priming group. The representative electrodes for P1 and N1 component were O1, PO3, PO7, O2, PO4, and PO8.

**Fig 5:**
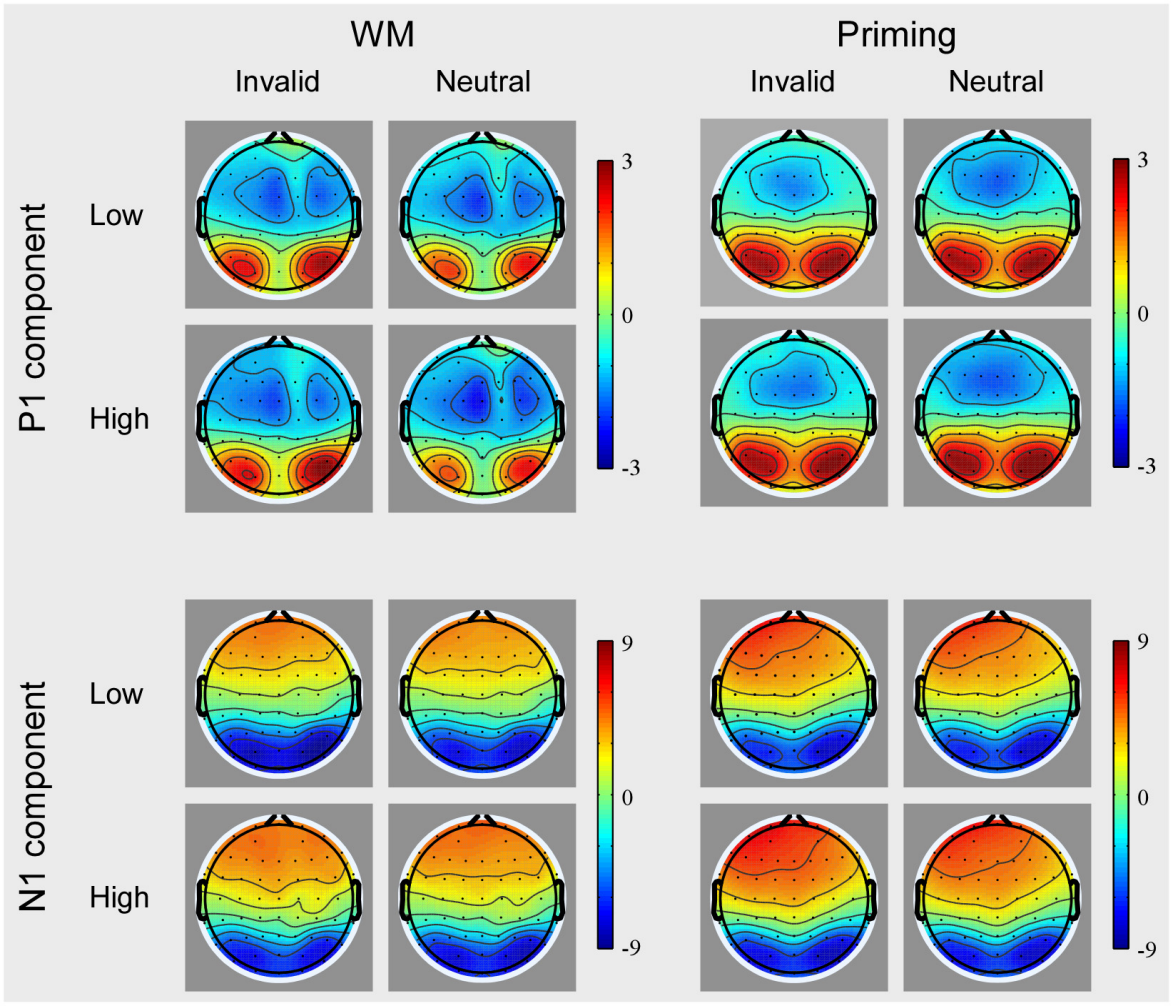
The scalp voltage distribution maps of the P1 (upper panels) and N1 (lower panels) component as a function of perceptual load and validity when the cue was held in WM (left column) and when it was merely identified (right column). The time points of the P1 and N1 components were 80–120 ms and 150–190 ms both in the WM group and the mere-repeat group, respectively. The color bars show the voltage value (in μV) of the component.

A four-way ANOVA with group (WM, priming), load (low, high), validity (invalid, neutral), and hemisphere (ipsilateral, contralateral) was conducted on the P1 amplitude. There was a reliable interaction effect between group and validity [*F* (1, 36) = 4.09, *p* < 0.05]. A further breakdown of interaction showed a reliable effect of validity in the WM group [*F* (1, 36) = 6.35, *p* < 0.05] and not in the priming group [*F* < 1]. The interaction between group, load and validity was not significant [*F* < 1.09]. In WM group, the validity effect was significant both at low perceptual load [*F* (1, 17) = 3.49, *p* < 0.05] and high perceptual load [*F* (1, 17) = 3.72, *p* < 0.05; see Figs 3 and 4A]. However, in the priming group, neither low perceptual load nor high perceptual load has significant validity effect (*F*s < 1.13; see Figs 3 and 4B). The scalp distribution of the P1 component is shown in Fig 5. No significant main effects or interactions were observed on the P1 latency.

#### N1 component

A four-way ANOVA with group (WM, priming), load (low, high), validity (invalid, neutral), and hemisphere (ipsilateral, contralateral) as within-subjects variables was conducted on the N1 amplitude. The results indicated that the main effect of perceptual load was significant [*F* (1, 36) = 22.03, *p* < 0.001], suggesting that high perceptual load trials elicited a more negative N1 amplitude (-9.13 ± 0.76) compare to low perceptual load trials (-8.44 ± 0.80). Crucially, there was also a reliable interaction between group, perceptual load and validity [*F* (1, 36) = 4.46, *p* < 0.05]. A further breakdown of the interaction showed a reliable interaction effect between perceptual load and validity in the WM group [*F* (1, 17) = 4.58, *p* < 0.05; see Figs 3 and 4C] and not in the priming group [*F* < 1.02; see Figs 3 and 4D). Specifically, in WM group, the validity effect on N1 amplitude (invalid minus neutral) was significantly present at low perceptual load [*F* (1, 17) = 4.97, *p* < 0.05], not at high perceptual load [*F* < 1]. The scalp distribution of the P1 component is shown in Fig 5. For the N1 latency, no significant main effects or interactions were observed.

### Source localization

**Fig 6:**
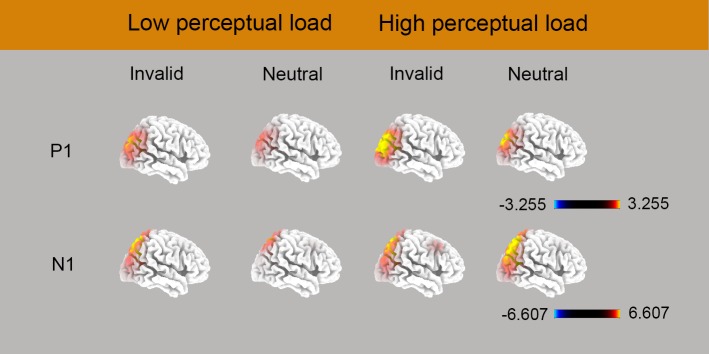
Displays grand mean sLORETA images of P1 (80–120 ms) and N1 (150–190 ms) for the four conditions in the WM group. Color bars represent voxel current density values (A/m2).

Estimates of the underlying cortical generators obtained using sLORETA are displayed in Fig 6. As shown in the figure, among four conditions for WM group, P1-related activation was located at superior occipital gyrus (BA 19, peak activation 35, -85, 30). For low-load match condition and high-load match condition, N1-related activation was located at precuneus, an area in parietal lobe, for (BA7, peak activation -10, -65, 65 for low-load match condition; peak activation -10, -60, 55 for high-load match condition). Under low-load mismatch condition and high-load mismatch condition, the maximum N1-related activation was located respectively at superior parietal lobule and inferior parietal lobule (BA7, peak activation -15, 65, 60 for low-load mismatch condition; BA40, peak activation 30, -60, 45 for high-load mismatch condition). All of the above activations have a bilateral feature, only the coordinates of area with maximal activation was reported.

## Discussion

To our knowledge, this is the first ERP study to explore the time course of the interaction between perceptual load and WM guidance on visual selective attention. The behavioural data showed that the WM guidance effect was present at low perceptual load condition, but not at high perceptual load condition. By contrast, there were no behavioural effects when the cue was identified but not held in memory. Futher, the ERP results showed that, like the effect on RTs, ERP effect was apparent only in the WM group. The P1 amplitude was increased when the WM stimulus reappeared as the distractor in visual search, at both low- and high-perceptual load condition, reflecting WM guidance effects. Nevertheless, in the N1 component, this initial WM guidance effect was absent at high perceptual load condition, and still present at low perceputal load condition. The earliest ERP modulation of WM guidance effect may be reflected on the P1 component. The larger amplitude in the P1 time range (80–120 ms) for invalid stimuli relative to neutral stimuli was a strong evidence of WM guidance effect. In fact, with a simple visual search task, we had found an incresed P1 amplitude effect when the WM stimulus reappeared in the visual search task [25]. Here, we extended this finding by showing that WM guidance effect was still significantly present even though the perceptual load was high during the visual search. Indirect comparisions between these two studies which used similar stimuli suggest that the earliest WM guidance effect has a preferential effect on selection relative to the influence from the level of perceptual load.

P1-related sLORETA results suggested that the strongest WM guidance effect occurred in the occipital area, which is in accordance with recent some fMRI studies[28,41,42]. These evidences suggest that the feature WM-based network involves the fronto-temporal-occipital regions, and the reapprearance of a stimulus held in WM enhances activity in those areas [28,42]. After a visual object is represented in WM, neurons in the prefrontal cortex carry signals related to the WM representation and feed back to the visual cotex to enhance the activity of the neurons that code memory-relevant features and promote the selection of matching viusal items during later search [41]. We suggest that the increased P1 amplitude in temporal-occipital area for memory-matching distractor reflects feedback from higher-order brain cortex, such as prefrontal cortex. With the feedback mechanism, visuospatial attention may enhance the perceptual salience of the memory-matching element from view field, which in turn results in the earliest WM guidance effect.

Importantly, the interaction between WM guidance and perceptual load was reflected on the N1 component. Similar to the pattern of behavioral results, at low perceptual load condition, the N1 amplitude on invalid trials was larger when the WM stimulus was repeated during search task relative to when it was not; however, this effect was eliminated when perceptual load was high. Given that N1 amplitude reflects the difficulty of target discrimination [43], these results suggested that perceptual load modulates attentional selection at an early processing stage, but following percepual discrimination [44]. Khoe, Freeman, Woldorff, and Mangun (2006) [45] also supported that the distractor interacted with target processing at a relatively late processing stage (between 180 and 250 ms). In the current study, the interaction was only significant for the N1 component, but not for the P1 component, akin to the other perceptual load related attention studies [13,15,38]. This finding suggests that the level of perceptual load is an important factor determining how information in WM influences attention.

The current N1-related effect was localized by sLORETA to the brain areas around the parietal lobe, consistently with previous N1 source data [46,47] and a recent ERP study [15], in which the activation for pereputal load by attention interaction was observed in TPO (temporoparietal-occipital gyrus). An fMRI study also suggested that the parietal lobe is involved in the integration of relevant feature- and space-based cues to optimize the deployment of attention in visual search [48] and is also critical for strategic modulation of WM biases through expectations/foreknowledge about the incoming validity of WM items for visual selection goals, namely, boosting or suppressing WM biases when WM contents predict a target or a distractor [49]. In addition to control of WM biases, some other studies further illustrated the parietal cortex may also play a role in generating a “template for rejection” [50] that may help to prevent the attention from capturing salient items [51] or WM content [29,49,52]. Based on above findings, the present parietal-localized N1 component should reflect the suppression processing to distractor and discrimination process to target. Specifically, the high-load stimuli in the visual search task may have required a higher level of discrimination processing and few processing resources will be available to be deployed to process distractors [1]. In the situation of low perceptual load, however, any capacity not taken up in perceiving task-relevent stimuli resulted in the the further processing for distractors. Therefore, in order to search the target successfully, participants have to suppress the interference from memory-matching distractor, reflected by larger N1 amplitude in invalid condition compare to neutral trials [25].

Why does perceptual load interact with WM guidance effect at an early processing stage as we saw with N1, but not upon the earlier P1 component? According to the capacity theory [13], whether the load effect on attention is upon the P1 or N1 component is determined by the relationship between the perceptual load and the available attentional resources indexed by each component. Their results demonstrated that the P1 has smaller capacity limit than the N1. Thus, P1 was not affected by perceputal load because it had exceeded capacity limits even when load was low. However, in the present results, we can not provide a quantitative evaluation of the absolute resource required by low- and high-perceptual load stimuli. So it is hard to predict whether the perceptual load exceeds the P1 resource limit or not. Consideration of a higher level of discrimination processing in high-load stimuli in the present study [43,53], with larger N1 amplitude in high perceptual load condition compare to low perceptual load condition, the perceptual load interacted with WM guidance was present at a relatively late processing stage (approximately 150–190 ms). Furthermore, note that the influence of load on spatial attention depends on whether or not subjects can anticipate the load of an impending target [14], P1 effects should be eliminated if load is randomly varied within blocks [15]. In our design, low- and high perceptual load conditions were not blocked but were mixed within the same blocks. It is speculated that an earlier perceptual load by attention interactions would be observed when perceptual load is held constant across trial blocks.

However, there was no electrophysiological evidence for bottom-up priming in the current study, which seems to be in disagreement with the fMRI study by Soto, Humphreys and Rotshtein (2007) [28]. Theyhas shown that mere stimulus repetition elicited a suppressive response in superior frontal gyrus, midtemporal and occipital areas. In contrast, the reappearance of a stimulus held in WM enhanced activity in the same regions. Strikingly, however, the mere-repetition effect was not found in ERP study [25,36]. Kumar, Soto and Humphreys (2009) [36] speculated that priming effect may reflect faster perceputal processing of display but without a strong “drive” of attention. In our study, the priming stimulus was always distractor and peripheral, which is distinct from the target-relevant search and center of visual field. This feature may be weaken the priming effect.

In conclusion, the present work shows that when perceptual load and WM guidance simultaneously exert their influences on selective attention, information in working memory could prior to capture attention in the early stage of visual processing (indexed by P1 component), consistently with the biased competition model of visual selection [4]. Later, this initial capture of attention by WM was modulated by the level of perceptual load, relected by the absolutely disappeared WM guidance effect with increased perceptual load (indexed by N1 component), consistently with the perceptual load theory [1,54]. Furthermore, WM guidance effect and perceptual load effect on the visual attention were localized into the temporal-occipital area and parietal lobe, respectively. The current results reveal the interaction between perceptual and WM guidance on the visual selective attention, and neural dynamics subserving this interaction.
